# Dual Energy Computed Tomography of Internal Carotid Artery: A Modified Dual-Energy Algorithm for Calcified Plaque Removal, Compared With Digital Subtraction Angiography

**DOI:** 10.3389/fneur.2020.621202

**Published:** 2021-02-04

**Authors:** Hongying Qu, Yongan Gao, Meiling Li, Shuo Zhai, Miao Zhang, Jie Lu

**Affiliations:** ^1^Department of radiology, Xuanwu Hospital, Capital Medical University, Beijing, China; ^2^Beijing Key Laboratory of Magnetic Resonance Imaging and Brain Informatics, Beijing, China; ^3^Department of Nuclear Medicine, Xuanwu Hospital, Capital Medical University, Beijing, China

**Keywords:** dual-energy computed tomography, internal carotid artery, calcified plaque, calcified stenosis, blooming artifacts, modified algorithm

## Abstract

**Background:** Atherosclerotic disease of the internal carotid artery (ICA) is a common reason for ischemic stroke. Computed tomography angiography (CTA) is a common tool for evaluation of internal carotid artery (ICA) stenosis. However, blooming artifacts caused by calcified plaques might lead to overestimation of the stenosis grade. Furthermore, the intracranial ICA is more vulnerable to calcification than other ICA segments. The proposed technique, dual-energy computed tomography (DECT) with a modified three-material decomposition algorithm may facilitate the removal of calcified plaques and thus increase diagnostic accuracy.

**Objectives:** The objective of the study is to assess the accuracy of the modified three-material decomposition algorithm for grading intracranial ICA stenosis after calcified plaque removal, with digital subtraction angiography (DSA) used as a reference standard.

**Materials and Methods:** In total, 41 patients underwent DECT angiography and DSA. The three-material decomposition DECT algorithm for calcium removal was applied. We evaluated 64 instances of calcified stenosis using conventional CTA, the previous non-modified calcium removal DECT technique, the modified DECT algorithm, and DSA. The correlation coefficient (*r*^2^) between the results generated by the modified algorithm and DSA was also calculated.

**Results:** The virtual non-calcium images (VNCa) produced by the previous non-modified calcium removal algorithm were named VNCa 1, and those produced by the modified algorithm were named VNCa 2. The assigned degree of stenosis of VNCa 1 (mean stenosis: 39.33 ± 19.76%) differed significantly from that of conventional CTA images (mean stenosis: 59.03 ± 25.96%; *P* = 0.001), DSA (13.19 ± 17.12%, *P* < 0.001). VNCa 1 also significantly differed from VNCa 2 (mean stenosis: 15.35 ± 18.70%, *P* < 0.001). In addition, there was a significant difference between the degree of stenosis of VNCa 2 and conventional CTA images (*P* < 0.001). No significant differences were observed between VNCa 2 and DSA (*P* = 0.076). The correlation coefficient (*r*^2^) between the stenosis degree of the VNCa 2 and DSA images was 0.991.

**Conclusions:** The proposed DECT with a modified three-material decomposition algorithm for calcium removal has high sensitivity for the detection of relevant stenoses, and its results were more strongly correlated with DSA than with those of conventional CTA or the previous non-modified algorithm. Further, it overcomes CTA's previous problem of overestimating the degree of stenosis because of blooming artifacts caused by calcified plaques. It is useful to account for calcified plaques while evaluating carotid stenosis.

## Introduction

The incidence of ischemic cerebrovascular disease has increased alongside the increasing numbers of patients with hypertension, hyperglycemia, and hyperlipidemia (1). It is a major cause of death and disability, and it seriously reduces patients' quality of life (2). Internal carotid artery (ICA) atherosclerotic disease is a common cause of ischemic stroke (3). The intracranial ICA has a narrower lumen than the extracranial ICA, and it is more prone to calcification (4). Thus, it might be more vulnerable to blooming artifacts generated by calcified plaque. Such blooming artifacts of calcification in the intracranial ICA might lead to overestimation of vascular stenosis. Recently, for patients with intracranial ICA stenosis, standard drug treatment has been the first-line therapy. However, ischemic cerebrovascular events still occur frequently in some patients. Invalid patients with symptomatic severe intracranial ICA stenosis are suitable for interventional therapy according to the Warfarin and Aspirin for Symptomatic Intracranial Disease (WASID) study. Thus, it is necessary to evaluate the degree of vascular stenosis so that the optimal treatment can be chosen. Further, choosing between different treatments according to the degree of vascular stenosis is important for patients' prognosis (5, 6).

Digital subtraction angiography (DSA) is the gold standard for evaluation of the degree of ICA stenosis, but it is invasive and has periprocedural risks (7). Compared with DSA, some other methods are less invasive and used more prevalently, such as transcranial color-coded duplex sonography (TCCD), magnetic resonance arteriography (MRA), and computed tomography arteriography (CTA). TCCD relies more on the experience and expertise levels of the sonographer. Hence, that method has low repeatability. Another non-invasive method, CE-MRA or TOF-MRA, is limited by contraindications such as claustrophobia and cardiac pacemakers, and it takes a long time to perform. In addition, patients complicated with dysphoria cannot stay still for a long time during scanning, so it is also not suitable for such patients. Besides, TOF-MRA has low spatial resolution and high vulnerability to artifacts caused by blood flow (8). Another widely accepted technique for evaluating ICA stenosis is CTA, which has the advantages of three-dimensional volumetric data analysis, better visualization of distal arteries, and shorter time cost (9). The results of CTA correlate well with those of DSA, and have high diagnostic accuracy (3). However, CTA may be hampered by calcified plaques, which may cause blooming artifacts and lead to overestimation of vascular stenosis (10). Nevertheless, information about the degree of vascular stenosis can be used to select the best treatment for patients (5, 11).

Dual-energy CT (DECT) has been used with increasing frequency in recent years. This modality allows the simultaneous acquisition of low- and high-energy images in a single examination and thus avoids interscan motion. It also reduces the radiation dose and scan time (12). In previous research, the CT value of iodine increased much more than those of bone and calcification when the X-ray tube voltage was decreased (13, 14). Thus, DECT may also have the ability to distinguish between medium-contrast volumes and calcified plaques and then remove the influence of calcified plaques. Some studies have investigated the usefulness of DECT angiography for removing calcified plaques and bone (15). Uotani et al. performed plaque and bone removal to assess the degree of carotid stenosis and concluded that DE hard plaque removal is useful for the evaluation of ICA stenosis with calcification (16). Werncke et al. applied plaque and bone removal to assess the degree of stenosis of peripheral arteries. They observed that DE hard plaque removal is highly effective for heavily calcified plaques (17). However, when Thomas et al. (5) performed plaque and bone removal on 25 patients' images, the results indicated that although DECT had a stronger correlation with DSA than conventional CTA, it frequently overestimated the degree of stenosis. However, distinguishing the degree of stenosis is clinically necessary (5, 18). Thus, a modified technique is needed. Recently, Mannil et al. used a novel modified DECT material-differentiating algorithm to evaluate the degree of stenosis of the extracranial ICA and found that their algorithm removed calcified plaques more accurately (19). However, patients with severe stenosis were not included (19), and research only applied the modified method to detect stenosis of the extracranial ICA.

Therefore, the purpose of this study is to evaluate the accuracy of the novel modified DECT material-differentiating algorithm for detecting the degree of stenosis of the intracranial ICA.

## Materials and Methods

### Patients

A single-center, prospective trial was conducted from January 2018 to March 2019. We collected patients' data from the Department of Neurosurgery, and all patients' medical history was assessed. Physical examinations, laboratory testing, and imaging examinations were also conducted.

The inclusion criteria were patients with intracranial ICA stenosis who underwent both intracranial and extracranial DSA and DE-CTA, the intracranial ICA stenosis was caused by calcified plaque, and there was a <2-week interval between DSA and DE-CTA. The exclusion criteria were unstable clinical conditions. Each patient signed a written consent form, and this research conformed to the principles outlined in the Declaration of Helsinki. We acquired ethical approval from Xuanwu Hospital of Capital Medical University.

### CTA Data Acquisition and Postprocessing

The CTA data were acquired with a 128-row multidetector CT scanner (Somatom Force^®^, Siemens, Munich, Germany). All patients were scanned from the aortic arch to the supraventricular white matter. The scan parameters were tube voltages of 150 and 90 kV, automatic adjustment of tube current according to patient size, pitch 1, slice thickness of 0.75 mm, layer spacing of 0.4 mm, rotation time of 0.5 s, FOV of 19–22 cm, and matrix size of 512 × 512.

The enrolled patients received 65 ml of contrast (Ultravist 370^®^, BayerSchering Pharma, Berlin, Germany) at a flow rate of 5 ml/s. The precise timing of the injection was determined using a test-bolus technique.

The conventional mixed CTA images were reconstructed with a weighting factor of 0.5, which resulted in a mixture of images from the 150- and 90-kV scans that resembled single-energy CTA. Then, the CTA data were transferred to a workstation (syngofastView^®^, Siemens Healthineers, Erlangen, Germany) for postprocessing. Virtual non-calcium (VNCa) images were generated according to the previous non-modified calcium removal DECT technique (VNCa 1) and the modified DECT algorithm (VNCa 2) using dedicated prototype software (eXamine, Version 0.9.10; Siemens).

### Qualitative Evaluation

Two experienced radiologists evaluated the results, and each was blind to the other's results. The image quality of conventional CTA and VNCa images was assessed on the following scale: 1 = perfect; 2 = not perfect, but could diagnose; 3 = non-diagnostic.

### Quantitative Evaluation

The stenosis measurements of the intracranial ICA on DSA, conventional CTA, and VNCa images were evaluated according to the criteria established by the North American Symptomatic Carotid Endarterectomy Trial. The formula: (the diameter of the normal artery beyond the stenosis–the diameter of the narrowest lumen)/the diameter of the normal artery beyond the stenosis × 100%.

Mean intraluminal attenuation values (Hounsfield units, HU) were measured in matched locations on conventional CTA and VNCa 2 images using a standardized circled region of interest. Two different experienced radiologists evaluated the DSA results, and they were blind to the CTA results.

### Statistical Analysis

Continuous variables were expressed as means ± standard deviations, and categorical variables were expressed as frequencies or percentages. Differences between stenosis measurements on conventional CTA, VNCa, and DSA were tested using analyses of variance. The differences in mean intraluminal attenuation values between VNCa 2 and conventional CTA were compared by a paired-samples *t*-test. Then, Pearson correlation was used to assess the relationship between DSA and VNCa 2. *P* < 0.05 were considered significant. Intrareader and interreader agreement regarding the degree of stenosis and qualitative imaging parameters was determined by intraclass correlation coefficients and Goodman and Kruskal's gamma, respectively.

## Results

This prospective study included data from 41 patients (29 men, 12 women) with 64 instances of calcified plaque on the intracranial ICA. Detailed information is found in Table 1.

**Table 1:** Descriptive statistics.

			***P***
Mean age, years	ALL	56 ± 7	
Sex	Male	29 (70.73%)	
	Female	12 (29.27%)	
Side	Left side	33 (51.56%)	
	Right sidetab	31 (48.44%)	
Good diagnostic image quality (%)	Computed tomography angiography (CTA)	62 (96.88%)	
	Virtual non-calcium images produced by the previous non-modified calcium removal algorithm (VNCa 1) and 2	62 (96.88%)	
Mean attenuation, HU	CTA	435.30 ± 83.60	0
	Virtual non-calcium images produced by the modified algorithm (VNCa 2)	399.37 ± 84.24	
Mean stenosis, %	CTA	59.03 ± 25.96	0.001
	VNCa 1	39.33 ± 19.76	
	VNCa 2	15.35 ± 18.70	
	Digital subtraction angiography (DSA)	10.78 ± 2.44	

### Qualitative Analysis

The image quality of the conventional CTA and VNCa was almost perfect. Only one patient's image with two instances of calcified plaque on the intracranial ICA was not perfect, and that result was influenced by a metal artifact. So in the next analysis, we removed the patient's data.

### Quantitative Analysis

**Figure 1:**
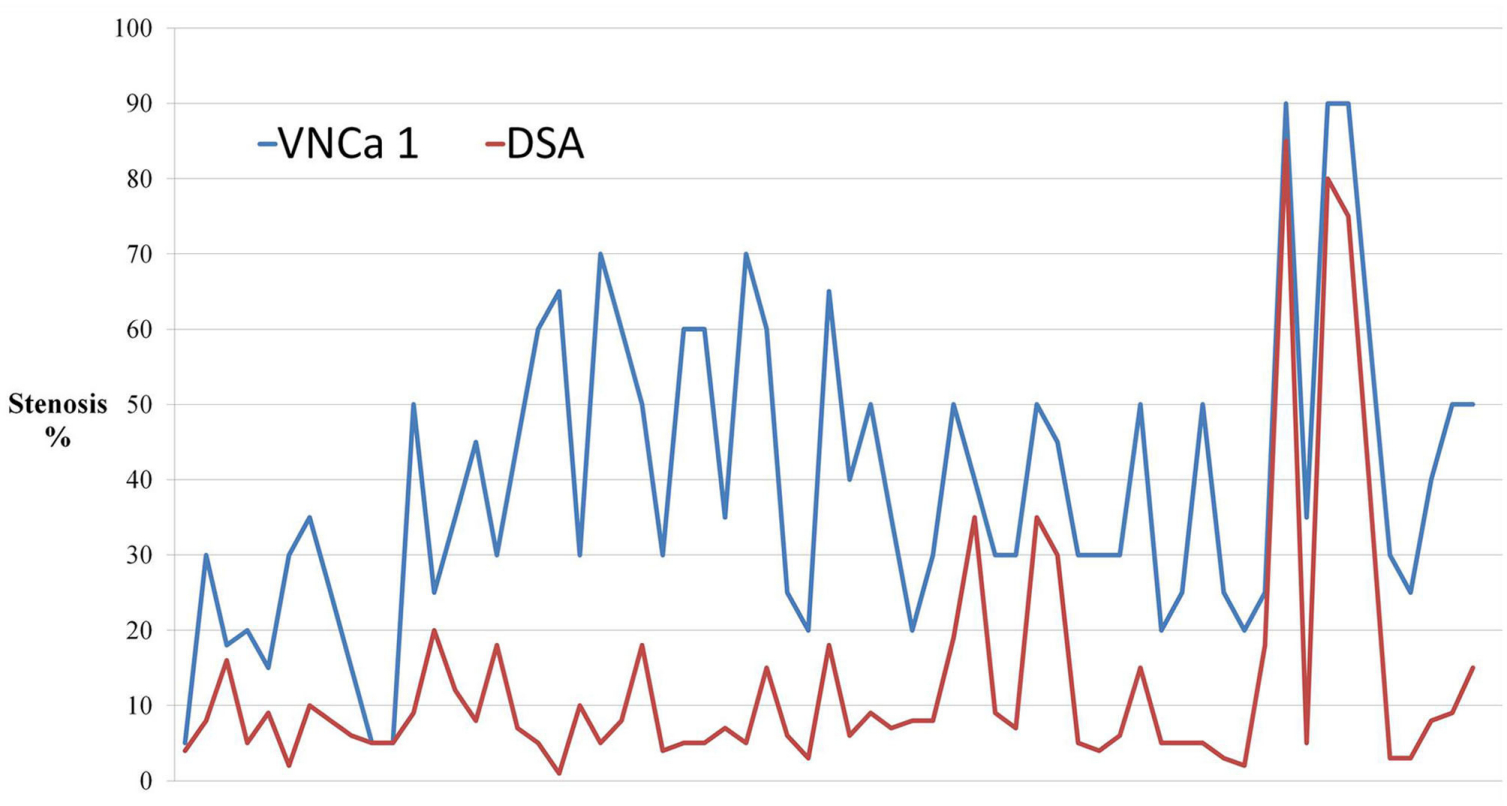
The dot-line graph showed the degree of calcified stenosis in virtual non-calcium images produced by the previous non-modified calcium removal algorithm (VNCa 1) tend to overcome the degree of calcified stenosis compared with digital subtraction angiography (DSA).

**Figure 2:**
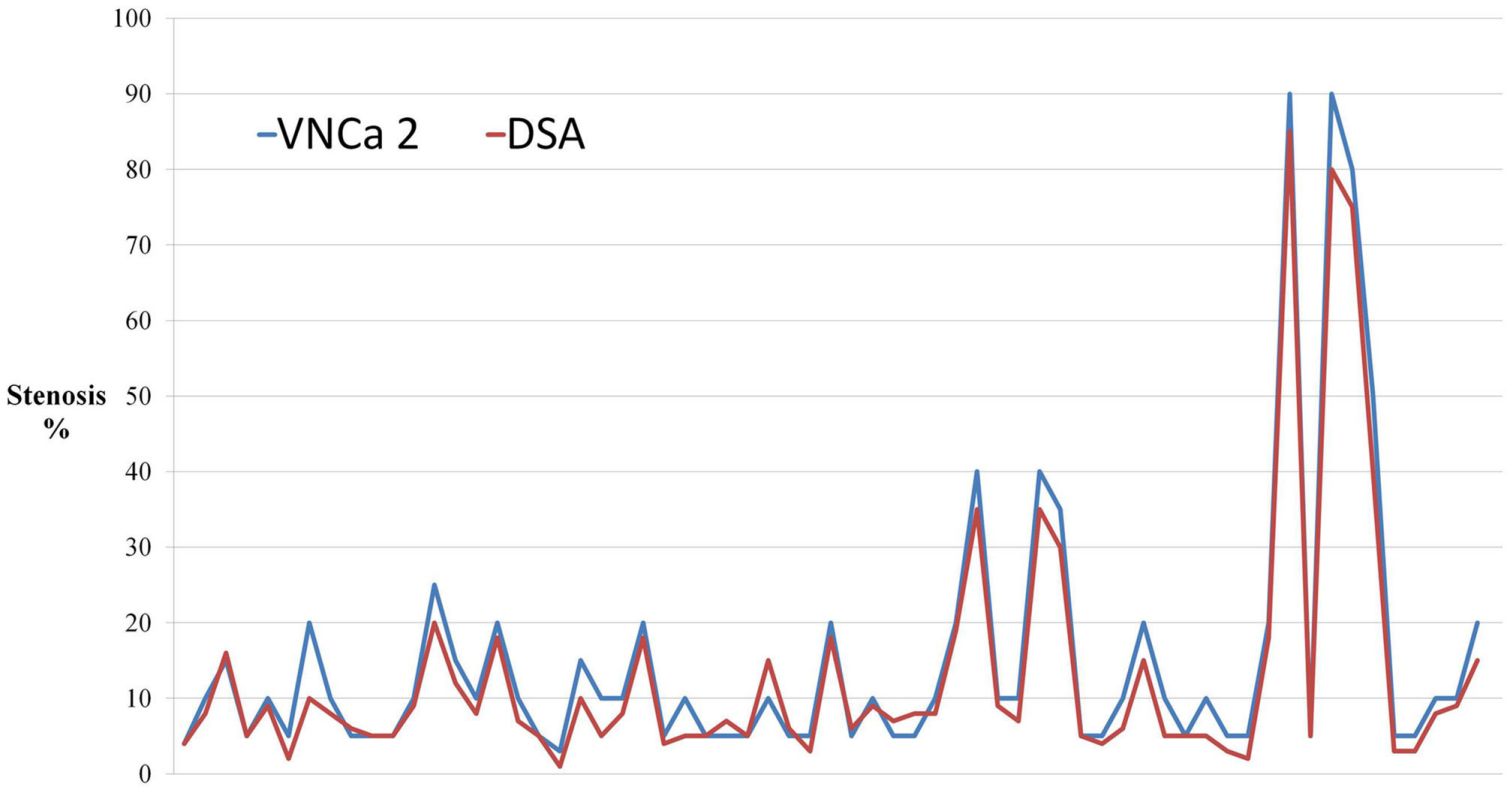
The dot-line graph showed that virtual non-calcium images produced by the modified algorithm (VNCa 2) had good consistency with DSA. Novel three-material decomposition dual-energy computed tomography (DECT) algorithm improves the diagnostic accuracy of computed tomography angiography (CTA).

**Figure 3:**
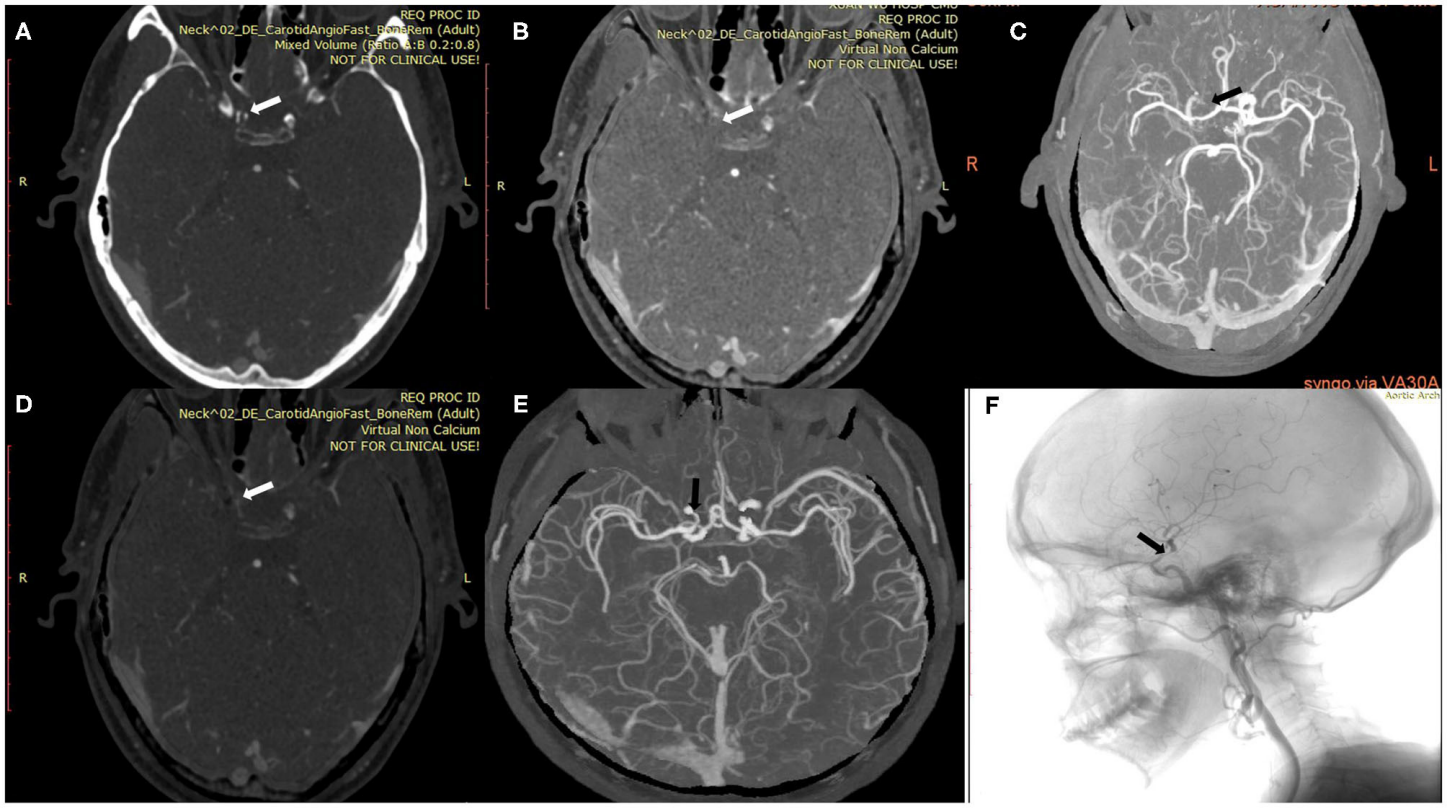
A 54-year-old man with calcified severe stenosis on the right intracranial internal carotid artery (ICA). **(A)** Axial conventional CTA at the location of calcified carotid artery stenosis (arrow). **(B,C)** Axial VNCa 1 and MIP at the location of calcified carotid artery stenosis (arrow). **(D,E)** Axial VNCa 2 and MIP at the location of calcified carotid artery stenosis (arrow). **(F)** DSA in sagittal projection indicated the severe stenosis (arrow). VNCa 2 had good consistency with DSA, and VNCa 1 tend to overcome the severe stenosis to occlusion.

**Figure 4:**
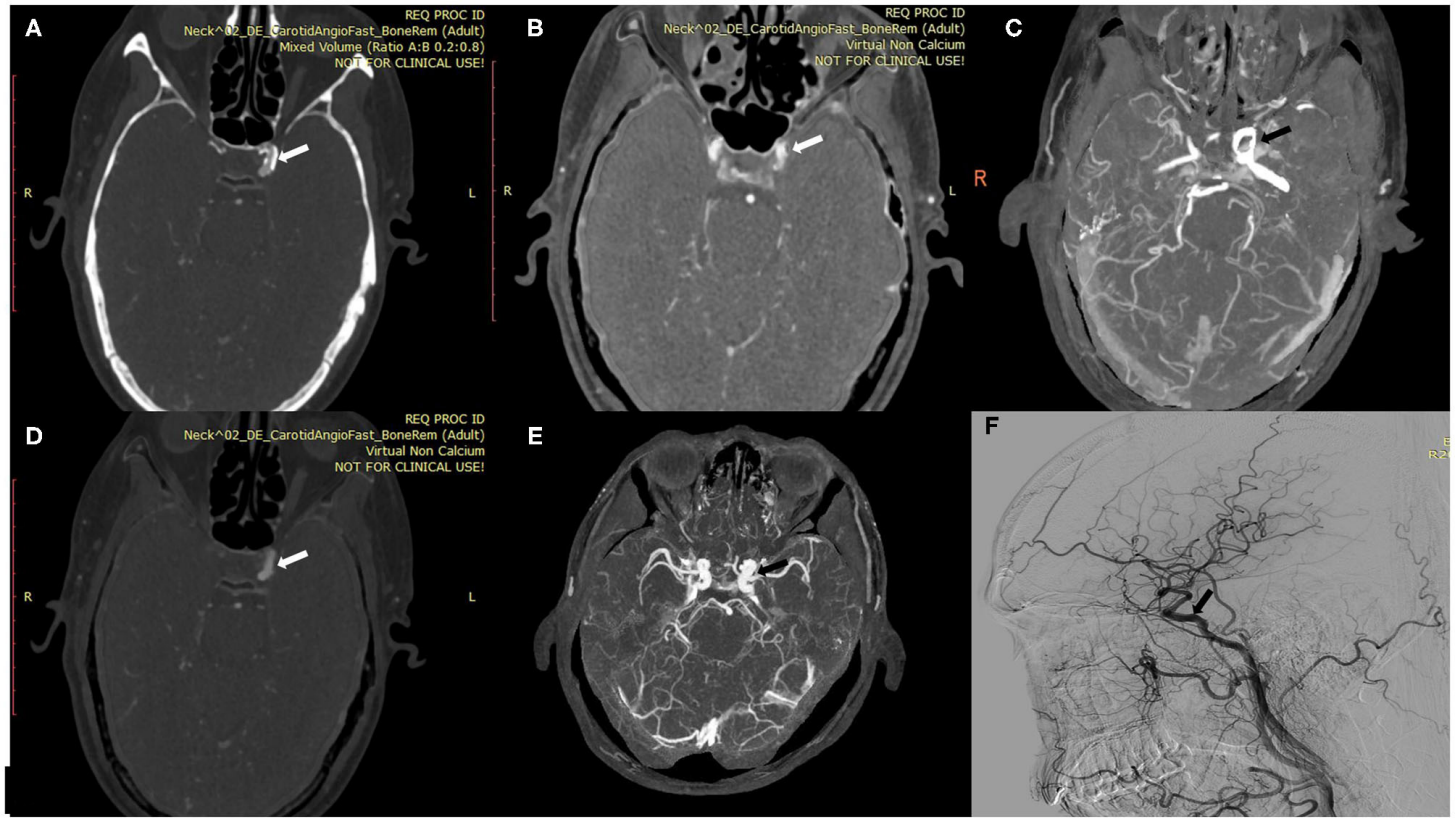
A 60-year-old man with calcified plaque on the left intracranial ICA. **(A)** Axial conventional CTA at the location of calcified plaque (arrow). **(B,C)** Axial VNCa 1 and MIP at the location of calcified carotid plaque (arrow). **(D,E)** Axial VNCa 2 and MIP at the location of calcified plaque (arrow). **(F)** DSA in sagittal projection indicated the no stenosis at the location of calcified plaque (arrow). The VNCa 1 still had residual calcification, and it tends to overcome the degree of calcified stenosis. Then VNCa 2 had good consistency with DSA.

The mean levels of carotid artery stenosis in DSA, conventional CTA, and VNCa 1 and 2 images were 13.79 ± 17.12%, 59.03 ± 25.96%, 39.33 ± 19.76%, and 15.35 ± 18.70%, respectively. Significant differences in stenosis level were observed among these four methods (*P* = 0.001; Table 1). The VNCa 1 results showed significant differences from those of conventional CTA, DSA images. VNCa 1 also significantly differed from VNCa 2. In addition, the stenosis level in conventional CTA images was significantly different from that in VNCa 2 and DSA images. There was no difference in stenosis levels between VNCa 2 and DSA images (*P* = 0.076; Table 2, Figures 1, 2). Representative examples of calcified carotid stenosis in DSA, conventional CTA, and VNCa 1 and 2 images are depicted in Figures 3, 4.

**Table 2:** Comparison of CTA, VNCa, and DSA stenosis measurements.

**Stenosis 1, %**	**Mean 1 ± SD**	**Stenosis 2, %**	**Mean 2 ± SD**	**P (Stenosis 1 vs. 2)**
VNCa 1	39.33 ± 19.76	CTA	59.03 ± 25.96	0.001
		VNCa 2	15.35 ± 18.70	0.000
		DSA	13.19 ± 17.12	0.000
VNCa 2	15.35 ± 18.70	CTA	59.03 ± 25.96	0.000
		DSA	13.19 ± 17.12	0.076
CTA	59.03 ± 25.96	DSA	13.19 ± 17.12	0.000

The mean intraluminal attenuation on conventional CTA images was 435.3 ± 83.6 HU, and the corresponding value at matched locations on VNCa 2 images was 399.37 ± 84.24 HU. The mean intraluminal attenuation on VNCa 2 images was significantly lower than that on conventional CTA images (*P* < 0.001; Table 1).

We selected four examples of DSA images for stenosis quantification, and they were evaluated as 30, 90, 75, and 50% stenosis. The same four lesions were quantified as 70, 100, 90, and 70% stenosis, respectively, on conventional CTA images; 50, 100, 90, and 50%, respectively, on VNCa 1 images; and 40, 90, 80, and 50%, respectively, on VNCa 2 images (Table 3). Among these lesions, conventional CTA and VNCa 1 tended to overestimate severe stenoses as occlusions or overestimate mild stenoses as moderate–severe stenoses. However, VNCa 2 and DSA had good consistency. The correlation coefficient (*r*^2^) of stenosis grading between the VNCa 2 and DSA images was 0.991.

**Table 3:** Patients with moderate to severe stenosis on DSA.

**Patients**	**1**	**2**	**3**	**4**
Age, years	49	61	75	76
Sex	Male	Male	Male	Female
The location of severe stenosis	C6 segment of right ICA	C6 segment of right ICA	C6 segment of left ICA	C4 segment of Right ICA
Degree of stenosis on DSA(%)a	30	90	75	50
Degree of stenosis on CTA(%)a	70	100	90	70
Degree of stenosis on VNCa 1(%)a	50	100	90	50
Degree of stenosis on VNCa 2(%)a	40	90	80	50

## Discussion

Our results indicate that the modified DECT algorithm is capable of removing calcified plaques and bone. Thus, it achieves improved quantification of intracranial ICA stenosis with DSA as the reference standard.

One of the innovations of DECT is that it overcomes mis-registration, which may occur in the conventional CTA method as a result of pulsations or neck movements during pre- and post-contrast scanning (20, 21). It has the advantages of low radiation dose and short scan time (22, 23), and it can remove the influence of calcified plaques and increase the accuracy of diagnosis of the degree of vascular stenosis.

Some studies have investigated the usefulness of the early DECT algorithm for removing calcified plaques and bone. For example, Uotani et al. (16) and Werncke et al. (17) applied DE plaque and bone removal to assess the degree of stenosis of carotid or peripheral arteries, finding that the technique was highly effective for heavily calcified plaques. However, some studies have also found that this technique has a disadvantage: it frequently leads to overestimation of the degree of vascular stenosis (5). Standard drug treatment is the first-line therapy for patients with intracranial ICA stenosis. However, some patients with severe intracranial ICA stenosis might fail to respond to medication, and interventional therapy could be suited for such patients, according to the WASID study. Further, interventional techniques may be unsuitable for patients with occlusion of the intracranial ICA. Hence, distinguishing the degree of stenosis is clinically important to choose suitable treatments for patients (5). Because of the above disadvantage, Mannil et al. applied a modified DECT algorithm to evaluate stenosis in the extracranial ICA and indicated that this new algorithm might improve the technique's accuracy (19). However, that study neglected to investigate intracranial ICA stenosis and did not compare the modified DECT algorithm with previous calcium removal DECT techniques.

In the present study, we applied this modified DECT algorithm to evaluate the degree of stenosis of the intracranial ICA. The intracranial ICA has a smaller lumen than the extracranial ICA. The tortuosity of the intracranial ICA makes it more prone to calcification of the vessel walls (24, 25). The influence of blooming artifacts in the intracranial ICA is more obvious than that in the extracranial ICA. The intracranial ICA is located at the base of the skull. Sometimes, the skull base bone can also interfere with judgment of vascular stenosis (26). Our results indicate that the modified DECT algorithm is more accurate than conventional CTA and previous non-modified calcium removal DECT technique, and its results are strongly correlated with those of DSA. This modified DECT algorithm has the potential to effectively increase the clinicians' ability to test the degree of vascular stenosis. Besides, the previous non-modified calcium removal DECT technique had higher diagnostic accuracy than conventional CTA but also tended to overestimate the degree of vascular stenosis.

In addition, we enrolled some patients with moderate–severe stenosis of the intracranial ICA in this study, providing a necessary complement to previous studies. Distinguishing between severe stenosis and occlusion or between mild–moderate stenosis and severe stenosis is clinically important, as the treatments for those conditions are different. For example, occlusions are not appropriate for stenting. If treatable stenoses are categorized as occlusions, treatment could be denied to patients (5, 18). Further, interventional therapy is not recommended for mild–moderate intracranial ICA stenosis. Thus, the assessment of the degree of vascular stenosis could affect patients' prognosis. In this study, the modified DECT algorithm and DSA produced consistent results, but conventional CTA and the previous non-modified calcium removal DECT technique tended to overestimate vascular stenosis. Thus, the modified DECT algorithm could improve the clinicians' diagnostic ability.

In this study, the modified DECT algorithm successfully removed calcified plaques from the intracranial ICA in almost all cases (96.88%). Only two calcified plaques were removed insufficiently (i.e., large residual calcifications were present). Serious metal artifacts might lead to undesirable results. Also, the level of intraluminal attenuation on VNCa 2 was significantly lower than that on conventional CTA images, indicating that the modified DECT calcium removal algorithm could partially remove iodine as well, which implies that the modified algorithm still has limitations in its current form.

Our study has the limitation of a small sample size. Larger trials need to be conducted to test our results.

The novel three-material decomposition DECT algorithm removed the calcified plaques on intracranial ICA in CTA images effectively and improved image quality. Its results were strongly correlated with those of DSA and overcame CTA's previous problem of overestimating degree of vascular stenosis. Thus, it has the potential to improve the diagnostic accuracy of CTA.

## Data Availability Statement

The raw data supporting the conclusions of this article will be made available by the authors, without undue reservation.

## Ethics Statement

The studies involving human participants were reviewed and approved by Xuanwu Hospital of Capital Medical University. The patients/participants provided their written informed consent to participate in this study. Written informed consent was obtained from the individual(s) for the publication of any potentially identifiable images or data included in this article.

## Author Contributions

HQ participated in the experimental design, data collection and post-processing, and paper writing. ML and SZ participated in the data collection and post-processing. JL not only provided the experimental equipment and post processing workstation but also revised the first draft of the paper. MZ participated in the data collection and giving a hand in the statistical analysis. YG participated in the experimental design, data post-processing, and revising of the paper. All authors contributed to the article and approved the submitted version.

## Conflict of Interest

The authors declare that the research was conducted in the absence of any commercial or financial relationships that could be construed as a potential conflict of interest.
